# A machine learning-based typing scheme refinement for *Listeria monocytogenes* core genome multilocus sequence typing with high discriminatory power for common source outbreak tracking

**DOI:** 10.1371/journal.pone.0260293

**Published:** 2021-11-19

**Authors:** Yen-Yi Liu, Chih-Chieh Chen

**Affiliations:** 1 Department of Public Health, China Medical University, Taichung, Taiwan; 2 Institute of Medical Science and Technology, National Sun Yat-sen University, Kaohsiung, Taiwan; 3 Rapid Screening Research Center for Toxicology and Biomedicine, National Sun Yat-sen University, Kaohsiung, Taiwan; Cornell University, UNITED STATES

## Abstract

**Background:**

As whole-genome sequencing for pathogen genomes becomes increasingly popular, the typing methods of gene-by-gene comparison, such as core genome multilocus sequence typing (cgMLST) and whole-genome multilocus sequence typing (wgMLST), are being routinely implemented in molecular epidemiology. However, some intrinsic problems remain. For example, genomic sequences with varying read depths, read lengths, and assemblers influence the genome assemblies, introducing error or missing alleles into the generated allelic profiles. These errors and missing alleles might create “specious discrepancy” among closely related isolates, thus making accurate epidemiological interpretation challenging. In addition, the rapid growth of the cgMLST allelic profile database can cause problems related to storage and maintenance as well as long query search times.

**Methods:**

We attempted to resolve these issues by decreasing the scheme size to reduce the occurrence of error and missing alleles, alleviate the storage burden, and improve the query search time. The challenge in this approach is maintaining the typing resolution when using fewer loci. We achieved this by using a popular artificial intelligence technique, XGBoost, coupled with Shapley additive explanations for feature selection. Finally, 370 loci from the original 1701 cgMLST loci of *Listeria monocytogenes* were selected.

**Results:**

Although the size of the final scheme (LmScheme_370) was approximately 80% lower than that of the original cgMLST scheme, its discriminatory power, tested for 35 outbreaks, was concordant with that of the original cgMLST scheme. Although we used *L*. *monocytogenes* as a demonstration in this study, the approach can be applied to other schemes and pathogens. Our findings might help elucidate gene-by-gene–based epidemiology.

## Introduction

With the increasing use of next-generation sequencing (NGS) to investigate pathogen genomes, gene-by-gene (GBG) approaches, including multilocus sequence typing (MLST) [1], whole-genome MLST (wgMLST) [2], and core genome MLST (cgMLST) [3], have become more frequently applied in genomic epidemiology [4]. cgMLST is the mainstream NGS-based typing method, and it has been successfully applied in the detection of outbreak clusters [5, 6]. Web services that work on cgMLST, such as PubMLST.org [7] and Pathogenwatch (https://pathogen.watch), have been supported by large companies and governments, indicating its significance. However, genomic sequences with varying read depths, read lengths, and assemblers influence the genome assemblies, which can introduce errors or missing alleles into the generated cgMLST allelic profiles, resulting in “specious discrepancy” among closely related or probably identical isolates. This, in turn, may influence outbreak interpretation. In addition, the rapidly growing number of cgMLST allelic profiles may cause problems with respect to storage and maintenance of the cgMLST allelic profile database.

In this study, by comparing the discrepancies among the profiles of cgMLST (1701 loci) and the LmScheme_370 (reduced cgMLST, 370 loci) generated from the 24 contigs, we observed that the error rate can be halved when using the reduced scheme. We also attempted to resolve the problems of specious discrepancy (i.e., using fewer loci to reduce the error rate), storage burden, and query search time by reducing the scheme size. The challenge of this approach is to retain the discriminatory power of the scheme to distinguish outbreaks with a greatly reduced typing scheme size. The solution is feature engineering, through which several algorithms can be employed for extracting essential features associated with specific classifications. *L*. *monocytogenes* was selected because it has ample data sources, especially two information-rich outbreak datasets [8, 9], for conveniently testing our proof of concept. The Extreme Gradient Boosting (XGBoost) coupled with the popular Shapley Additive exPlanations (SHAP) algorithm was used to reduce the size of the *L*. *monocytogenes* typing scheme [10]. To evaluate the applicability of our idea, we used XGBoost to train a dataset comprising 93 outbreaks of *L*. *monocytogenes* documented by Chen et al. [8] for feature selection and extracted the 370 most important loci. To evaluate the typing performance of the newly created scheme, an independent dataset comprising 35 outbreaks with more than one isolate and labeled cgMLST types (CT types) [9] was used.

Our approach effectively reduced the specious discrepancy, storage burden for maintaining allelic profiles, and query search time by reducing the scheme size through an artificial intelligence technique. Although we used *L*. *monocytogenes* in this study, the approach can be applied to other pathogens. Our findings might help elucidate some aspects of GBG-based genomic epidemiology.

## Materials and methods

In this study, 12 read sets simulated from three complete genomes of *Listeria monocytogenes*, introduced different read lengths and read depths, and applied two popular assemblers, SPAdes [11] and Skesa [12], were used for the assembling tasks, thus resulting in 24 contigs for demonstration. The *L*. *monocytogenes* cgMLST scheme published by Ruppitsch et al. [10] comprising 1701 loci was used because it is easy to download all the allele sequences of the scheme. To avoid bias, we used two *L*. *monocytogenes* datasets [8, 9] to generate loci selection (scheme reduction) and scheme evaluation. To extract important loci, the minMLST program implemented with the XGBoost algorithm was used for a dataset comprising 93 *L*. *monocytogenes* outbreaks as the training dataset (Set A, S1 Table). The subsequent evaluation was performed on an independent dataset comprising 35 *L*. *monocytogenes* outbreaks with more than one isolate (Set B, S2 Table).

### cgMLST scheme for *L*. *monocytogenes*

Although several cgMLST schemes exist for *L*. *monocytogenes*, such as those of Ruppitsch et al. [10] and Moura et al. [9], we selected the scheme published by Ruppitsch et al. [10] because the allelic sequences are easily downloadable (*cgMLST*.*org Nomenclature Server*, https://www.cgmlst.org/ncs/schema/690488/, hosted by Ridom https://www.ridom.de). The scheme comprises 1701 loci, and the alleles were obtained from 26,919 *L*. *monocytogenes* isolates on December 7, 2020. Allelic profiling and difference calculations among isolates were performed using the program from previous studies [13, 14].

### Locus reduction for the cgMLST scheme for *L*. *monocytogenes*

We selected XGBoost, an efficient supervised machine learning algorithm, to achieve locus reduction. In addition, we used minMLST [15], a package that implements the XGBoost algorithm and is specifically designed for cgMLST scheme reduction, and applied SHAP [16] values to select specific loci. All loci with SHAP values larger than zero were selected, and the best cluster tolerance cutoff value was obtained from the independent test set (Set B) by comparing the consistency of the total CT types included within each outbreak.

### Occurrence of specious discrepancy based on simulated reads with different experimental conditions

To demonstrate the occurrence of specious discrepancy, we downloaded the three latest deposited L. monocytogenes whole-genome sequences from NCBI Genome with completed assembly status: GCF_016775745.1, GCF_016802645.1, and GCF_905219385.1. To obtain different read lengths and read depths, we used ART [17], a popular NGS read simulator, to generate 20× and 50× Illumina MiSeq reads (250-bp read length) and 20× and 50× HiSeq reads (150-bp read length). The simulated read sets were assembled with SPAdes [11] and Skesa [12], two popular open-source assemblers. All the simulated data including read sets and assembled genomes can be downloaded from https://reurl.cc/MZYLvm.

### Training and evaluation dataset

The training data of Chen et al. [8] comprised 258 isolates belonging to 132 outbreaks. Only isolates with whole-genome sequencing (WGS) data in the NCBI SRA database were included. Hence, the final training set (Set A) comprised 159 isolates belonging to 93 outbreaks. All the genome data were downloaded from the NCBI Assembly and SRA databases. The isolates with raw reads obtained from the SRA database were quality trimmed and de novo assembled by Trimmomatic [18] and Shovill (https://github.com/tseemann/shovill), respectively. In addition, genomic assemblies downloaded from the Assembly database were quality checked with the tools CheckM [19] and fastANI [20]. Finally, N50, the metric typically used for assembly quality, was investigated. The independent evaluation data contained 322 isolates belonging to 44 outbreaks (of which 35 had more than one genome isolate) [9]. All the WGS download, quality control, and assembly procedures for Sets A and B were identical.

### Scheme performance evaluation

To evaluate the outbreak prediction performance of the reduced cgMLST (LmScheme_370) scheme, we designed an evaluation method in which the number of clusters (LmScheme_370 type) calculated from LmScheme_370 profiles was compared with CT types defined by Moura et al. [9] for each outbreak. The LmScheme_370 scheme predicted better performance, with fewer differences between the number of LmScheme_370 types and CT types. The Clusters of Orthologous Genes (COG) functional catalog predicted from the EggNOG mapper program [21] was also used for functional analysis of the final selected scheme.

## Results

The original cgMLST scheme for *L*. *monocytogenes* downloaded from the cgMLST.org Nomenclature Server comprised 1701 loci with 814,600 alleles. The reduced cgMLST scheme (LmScheme_370) contained 370 loci. In outbreak prediction, the highest accuracy obtained from the original and reduced cgMLST schemes was 83% (6 of 35) and 80% (7 of 35), respectively. The loci of LmScheme_370 belonged to 19 COG functional classes, with 13 loci with failed predictions and 17 loci predicted for more than one class. The tanglegram representation was used to demonstrate the comparison of the single-linkage dendrogram based on LmScheme_370 and the original cgMLST scheme for Set A; it revealed concordant clusters between the two schemes. For the independent test of LmScheme_370 in outbreak discriminatory ability, we used Set B, comprising CT types [9], as the model answer. The predicted accuracy of LmScheme_370 with Set B used for clustering the correct number of groups compared with CT types was 80% (28 of 35 outbreaks). The intraoutbreak maximum allelic distances based on both the original cgMLST and LmScheme_370 schemes were calculated, and revealed similar trends in Set B.

### Feature selection from XGBoost

**Fig 1:**
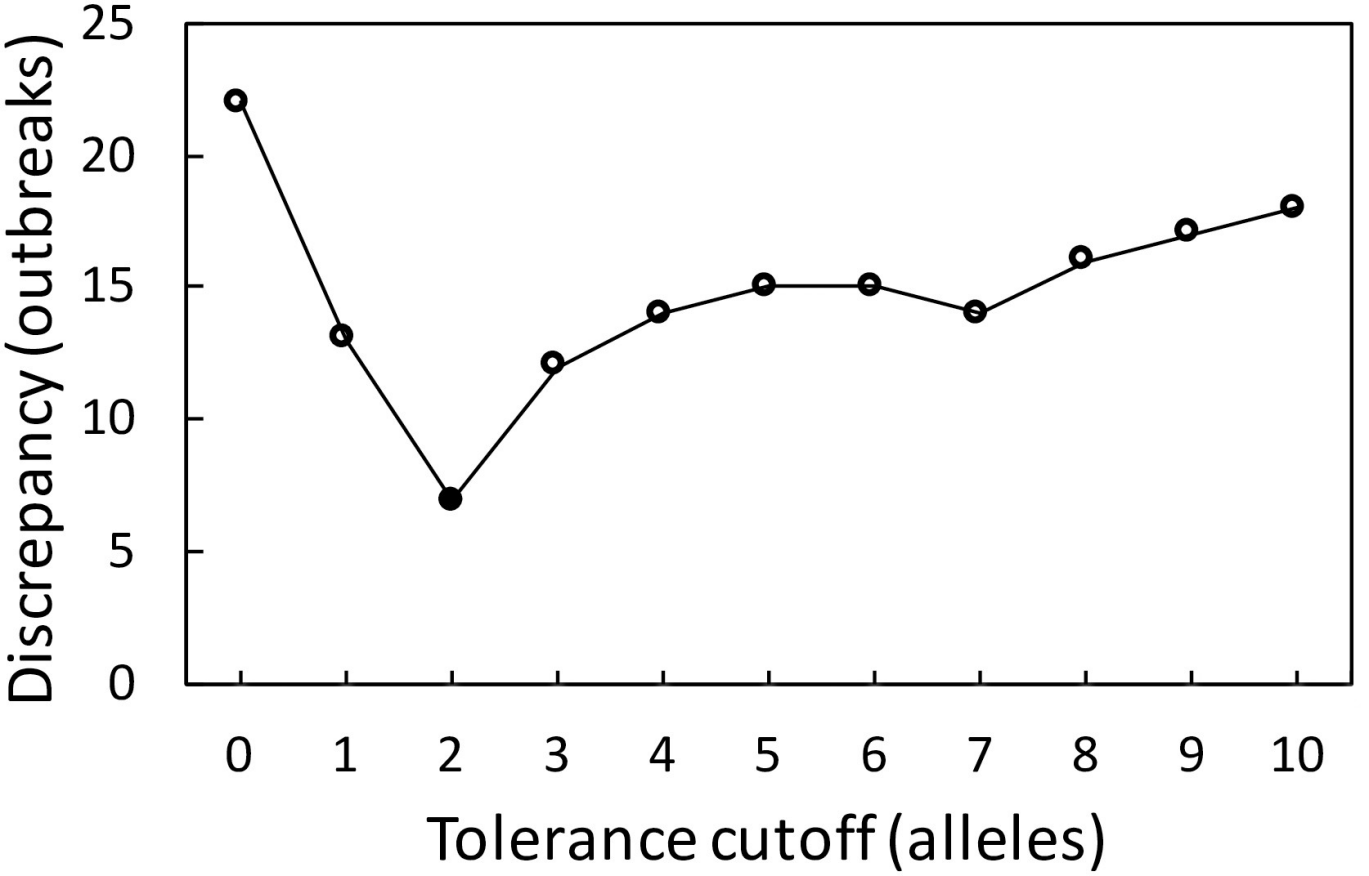
Discrepancy between CT types and LmScheme_370 types for the 35 outbreaks of Set B when the tolerance cutoff is varied from 0 to 10. The total numbers of different type counts in the 35 outbreaks of Set B between the CT types and groups counts based on the LmScheme_370 single-linkage clustering for each outbreak were calculated.

All loci with a SHAP value larger than zero were considered contributors to outbreak cluster prediction; thus, 370 of 1701 loci were selected for LmScheme_370 (S3 Table). Approximately one-third of the loci (106 loci) from LmScheme_370 were labeled with hypothetical proteins for their products (S3 Table), and two hypothetical loci were considered to be among the 10 most important loci. To determine the best cutoff value (tolerance of the allelic differences for the same outbreak cluster), we assessed 11 values from 0 to 10 for LmScheme_370. The total outbreaks with unconcordant counts of cgMLST types between CT and LmScheme_370 in 35 outbreaks of Set B were calculated. Fig 1 illustrates that the minimum difference had a cutoff value of 2 (i.e., allelic profiles with a difference of less than two alleles were considered to be in the same outbreak cluster). We therefore set the allelic difference cutoff value to 2 when applying LmScheme_370.

### Description of specious discrepancy based on simulated reads with different experimental conditions

The cgMLST (1701 loci) [10] allelic differences between the pairs of the assembled simulated genomes for the three demonstration *L*. *monocytogenes* isolates are presented in S4 Table. The maximum difference between assembled genomes with different simulated experimental conditions for each isolate could reach to more than 30 loci, which is much more than the allelic cutoff defined using outbreak data. The differences for each isolate were due to variations in read lengths, read depths, and assemblers and were therefore not the real discrepancies because they were the same isolates.

### Improvement of specious discrepancy using LmScheme_370

The recalculated allelic differences based on LmScheme_370 between pairs of the assembled simulated genomes for the three demonstration *L*. *monocytogenes* isolates are presented in S5 Table. The maximum difference between assembled genomes with different simulated experimental conditions for each isolate was less than 8 loci. Statistically, if we set two loci as the outbreak allelic cutoff for the LmScheme_370 scheme, 83% of simulated genome pairs were correctly identified as the same isolates compared with 68% for the Lm-cgMLST scheme with eight loci as the cutoff.

### Functional analysis of the loci in LmScheme_370

**Fig 2:**
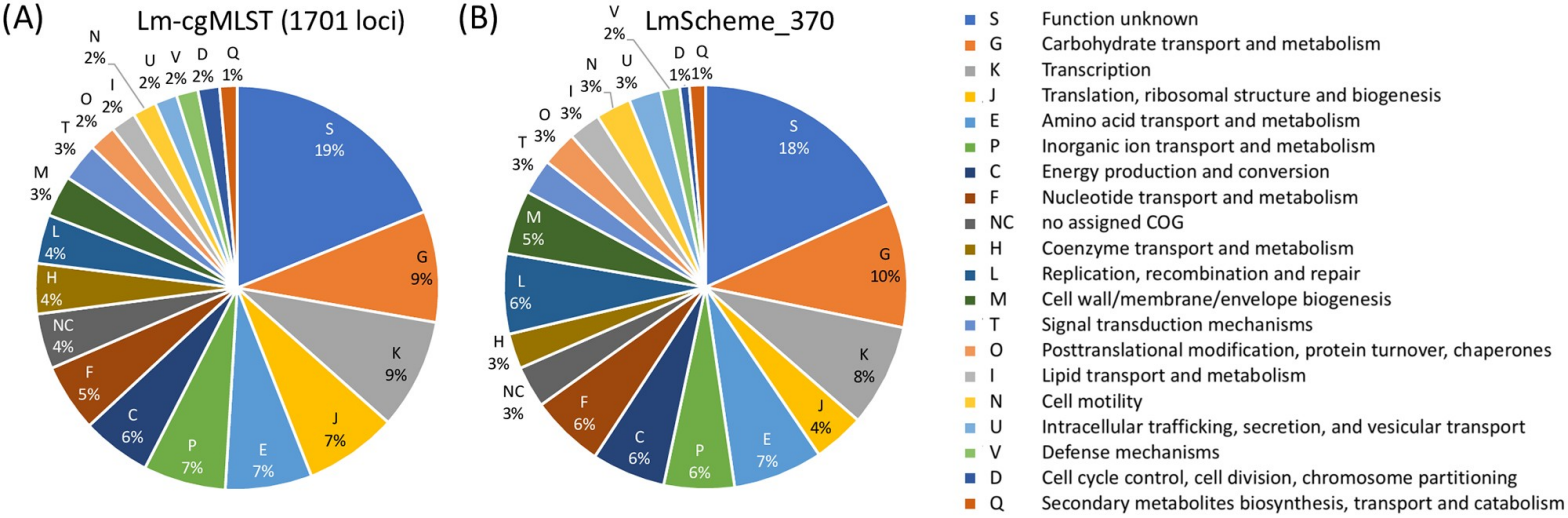
Distribution of COG functional classes for the loci of (A) Lm-cgMLST (1701 loci) and (B) LmScheme_370.

To understand the locus properties in LmScheme_370, we performed a functional analysis based on the EggNOG mapper program [21]. The COG functional catalog predictions show that 370 loci in LmScheme_370 belong to 19 functional classes (i.e., S, G, K, E, L, F, C, P, M, J, T, N, O, H, I, U, V, Q, and D), excluding 13 loci without a successful prediction (NC). A total of 17 loci were predicted to belong to more than one class. The total counts for each predicted functional catalog of LmScheme_370 are presented in Fig 2. Except for class S (function unknown), the five most abundant successfully annotated functions were G (carbohydrate transport and metabolism), K (transcription), E (amino acid transport and metabolism), L (replication, recombination, and repair), and F (nucleotide transport and metabolism). The COG functional distribution of the original scheme (1701 loci) is presented in Fig 2.

### Comparison of LmScheme_370 and cgMLST schemes for Set A

**Fig 3:**
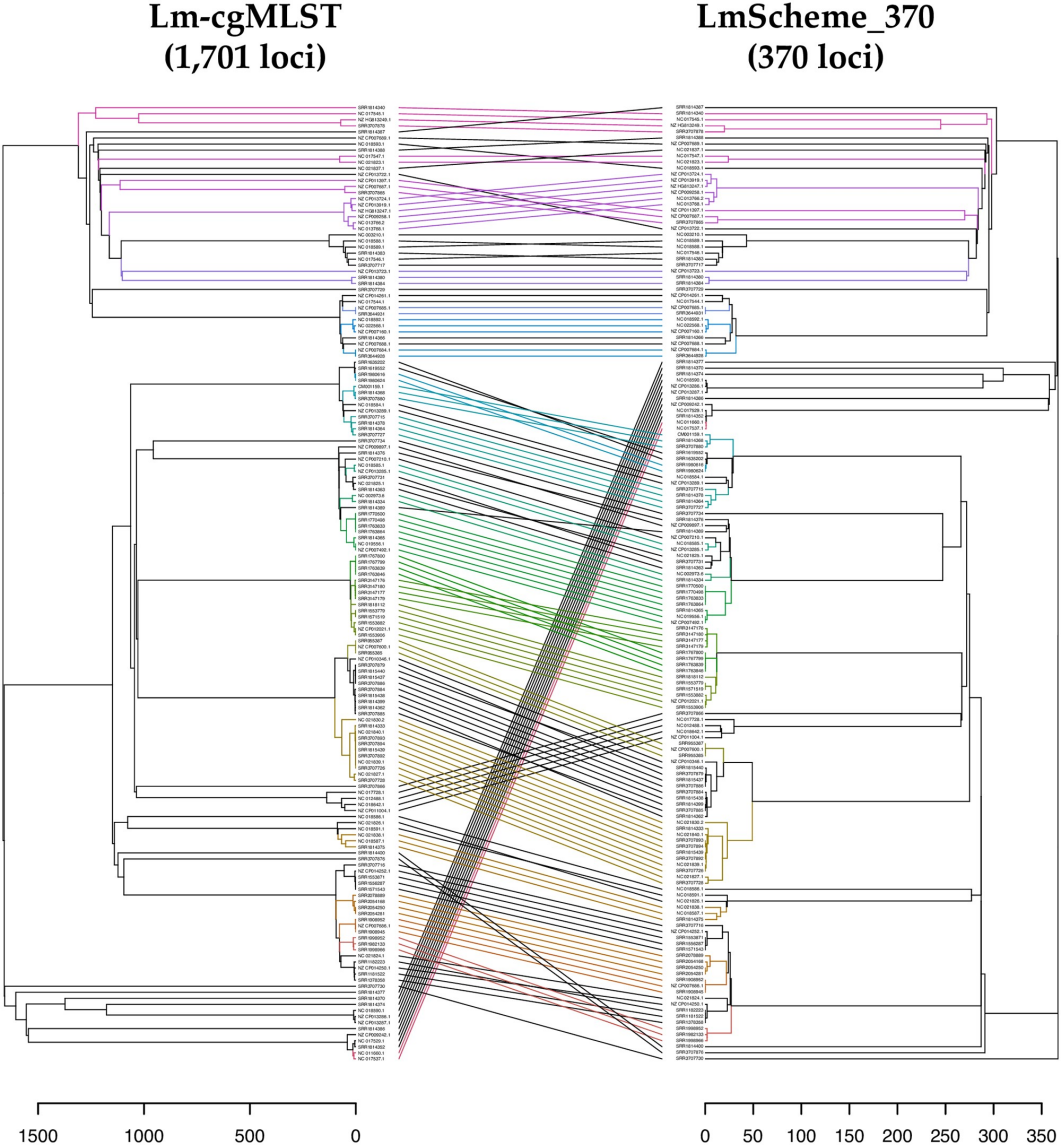
Tanglegram of dendrograms calculated based on Lm-cgMLST (1,701 loci) and LmScheme_370 allelic profiles from Set A. Both allelic distance scales are displayed. The same isolate in each dendrogram connected with a line; the colors have no meaning but are used for easier visualization.

Fig 3 illustrates the tanglegram comparing single-linkage dendrograms based on the core genome scheme (comprising 1701 loci) and LmScheme_370 (comprising the 370 most important loci) for the training dataset [Set A, from Pennsylvania State University collection [8]]. Although the tree topologies differed, the clusters were largely concordant, with only a few nodes rotated. This means that the cluster and outbreak detection abilities were conserved in LmScheme_370.

### Evaluation of the reduced *L*. *monocytogenes* cgMLST scheme based on an independent *L*. *monocytogenes* outbreak benchmark

**Fig 4:**
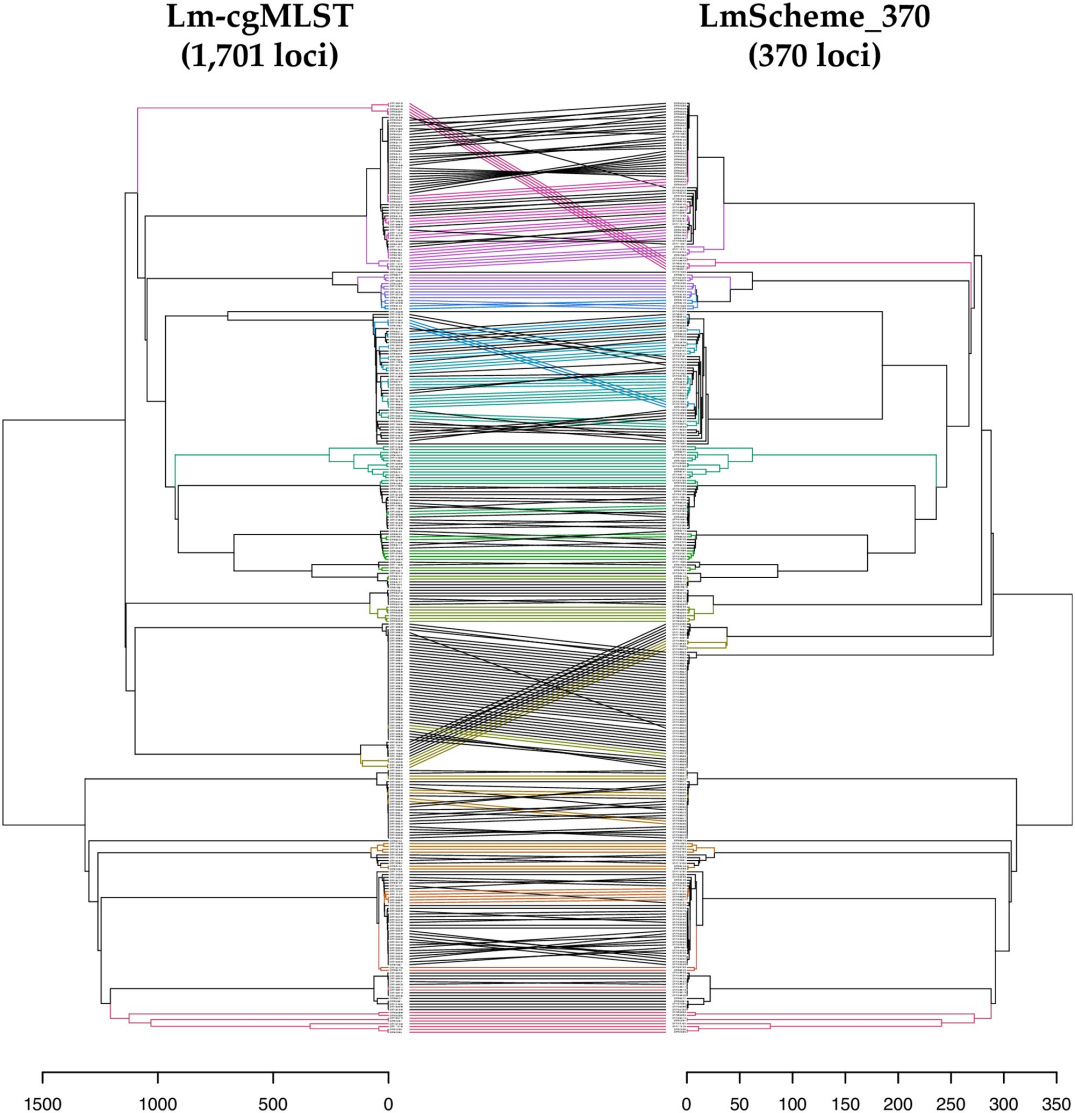
Tanglegram of dendrograms calculated based on Lm-cgMLST (1,701 loci) and LmScheme_370 allelic profiles from Set B (independent test set). Both allelic distance scales are displayed. The same isolate in each dendrogram is connected with a line; the colors have no meaning but are used for easier visualization.

The tanglegram for the single-linkage dendrogram of the independent dataset (Set B), comparing LmScheme_370 (comprising only the most important 370 loci) with the core genome scheme (comprising 1701 loci), is presented in Fig 4. The clusters in both schemes appeared concordant, with little displacement. A single-linkage dendrogram of Set B calculated based on LmScheme_370 with CT types and labeled outbreak clusters was also generated (S1 Fig). To further examine LmScheme_370, we compared the number of clusters (using the single-linkage method) calculated on the basis of LmScheme_370, with a cutoff tolerance of two alleles with the CT types (Table 1). Table 1 indicates that 7 of 35 outbreaks were dissimilar between the two schemes: outbreaks (1) 1307MLGX6-1, (2) 1309MLGX6-2WGS, (3) 1311MLGX6-1, (4) 1312LACGX6-1, cluster 5 for (5) *less groups defined* and (6) 1312MLGX6-1, and cluster 10 for (7) *more groups defined*, compared with CT types. The 1307MLGX6-1 outbreak was particularly different from CT types, with a seven-group discrepancy. The clusters with a two-allele tolerance discrepancy based on LmScheme_370 were concordant with the CT types [9], with only 7 of 35 outbreak clusters having dissimilar group numbers (predicted accuracy: 80%). The comparisons of LmScheme_370 types and CT types for each outbreak are presented in Table 1.

**Table 1:** The comparison of CT types and LmScheme_370 types for each outbreak in Set B.

Outbreak ID	CT types	LmScheme_370 types
0908MLGX6-1	1	1
1005NYGX6-1	1	1
1308MLGX6-2	1	1
MF-b	1	1
MF-c	1	1
MF-d	1	1
MF-e	1	1
Mom-a	1	1
cluster 10^#^	1	2
cluster 7	1	1
cluster 8	1	1
cluster 9	1	1
1207PAGX6-1	2	2
1307MNGX6-1	2	2
1308ILGX6-1	2	2
1308MDGX6-1	2	2
1309MLGX6-1	2	2
1312LACGX6-1*	2	1
1403MLGX6-1WGS	2	2
DK-CC224-2014	2	2
cluster 1	2	2
cluster 11	2	2
1308MLGX6-1	3	3
cluster 5*	3	1
1307MLGX6-1,2	4	4
1310VAGX6-1	4	4
1312MLGX6-1^#^	6	7
1411RIGX6-1WGS	6	6
1309MLGX6-2WGS*	9	8
1311MLGX6-2WGS	9	9
1311MAGX6-1	10	10
1307MLGX6-2	11	11
1311MLGX6-1*	11	9
1307MLGX6-1*	14	7
1301MLGX6-1	23	23

*Indicates the outbreaks that CT types are more than LmScheme_370 types.

^#^Represents CT types are less than LmScheme_370 types.

### Intraoutbreak and interoutbreak allelic number comparisons between the LmScheme_370 and cgMLST schemes

**Fig 5:**
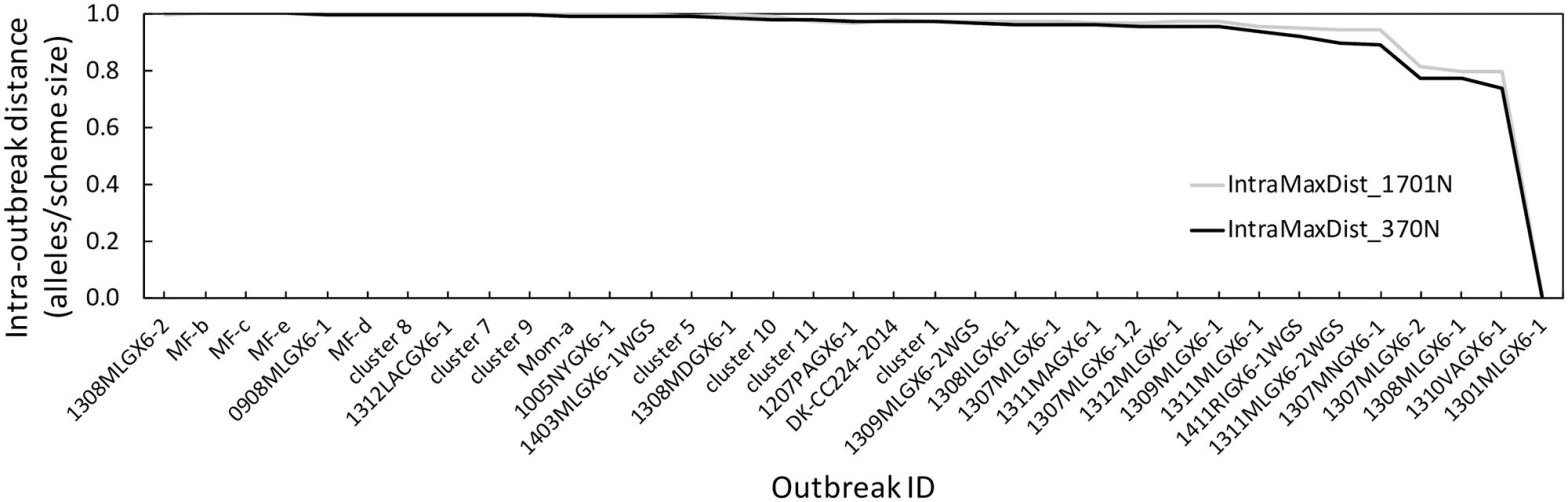
Maximum intraoutbreak allelic distance in the 35 outbreaks of Set B. The distance value is normalized by dividing by the scheme size (i.e., 1,701 for cgMLST and 370 for LmScheme_370). The black line indicates the distance calculated based on the LmScheme_370 scheme, and the gray line represents the distance calculated based on the Lm-cgMLST scheme.

To compare the clustering ability of LmScheme_370 with the original cgMLST scheme (1701 loci), we calculated the maximum allelic differences between isolates within the outbreak cluster for the 35 outbreaks in Set B. The maximum allelic distance was normalized by dividing the scheme size (i.e., 1701 for cgMLST and 370 for LmScheme_370) to obtain a comparable scale. The normalized distances for each outbreak (Fig 5) indicated similar intraoutbreak distances between the two schemes.

## Discussion

Genomic epidemiology in infectious disease monitoring and source tracking has become increasingly popular as WGS has become cost efficient. Among several sequence-based methods commonly used in genomic epidemiology, such as the whole-genome single-nucleotide polymorphism approach and GBG comparison methods, cgMLST (a GBG method) is the most commonly used. An allelic profile is generated, typically from thousands of loci predefined in the cgMLST scheme. By comparing the allelic profiles of the bacterial isolates, researchers can use their genetic relatedness to identify the source of infection and outbreak clusters. However, because the quality and experimental sequencing conditions of WGS vary for different isolates, erroneous or missing data might be incorporated into the generated allelic profiles. In addition, the assembler chosen can significantly influence the quality of genome assemblies and consequently the generated allelic profiles. In this study, we tested read depths of 20× and 50×, read lengths of 150 bp (HiSeq) and 250 bp (MiSeq), and two commonly used assemblers, SPAdes and Skesa. We also demonstrated how choosing different read lengths, read depths, and assemblers can incorporate erroneous and missing data into allelic profiles. The maximum allelic difference based on cgMLST (1701 loci) was observed between assemblies 20x_HiSeq_skesa and 20x_MiSeq_skesa (S4 Table), reaching more than 30 loci, with the average allelic distance larger than eight loci for the three isolates. The eight assembled genomes (based on different simulated conditions) for each isolate were all simulated from the same completed genome; therefore, they theoretically should have no discrepancy. As shown in S5 Table, the maximum allelic distance between assemblies with different experimental conditions of the three isolates was seven loci, with the average allelic discrepancy decreased to less than two loci for each isolate. In addition, when the average allelic discrepancy was considered the cutoff for each scheme (i.e., eight loci for LmScheme_370 and two loci for Lm-cgMLST) to ignore the difference, the specious discrepancy decreased from 32% to 17%. Although we used only three complete genomes for simulating reads with different sequencing conditions in this proof-of-concept study, the tendency for different isolates was similar; this implied that the specious discrepancy can be mitigated by reducing the scheme size. However, future studies should examine this approach with more data.

To reduce false discrepancies and decrease the storage burden, we reduced the size of the *L*. *monocytogenes* cgMLST scheme by approximately 80%, from 1701 to 370 loci. We were able to maintain the typing discriminatory power of the reduced scheme by using XGBoost, which is an excellent algorithm that can boost the feature selection step (i.e., select the most important loci that can distinguish the training outbreak dataset), to select the loci with large weights for discriminating between different outbreak clusters. COG functional analysis was conducted to predict these 370 loci. Except for the predicted hypothetical function, the most important loci were essential genes participating in biosynthesis and DNA replication. The percentage for each catalog was almost the same between the two schemes, meaning that the XGBoost could select loci from different catalogs in a balanced manner to generate the final reduced scheme. For clustering evaluation, which used the allelic profiles generated from only 370 loci, the tanglegram indicated that the two schemes were largely concordant. The prediction performance was tested more comprehensively by comparing the predefined CT types [9] for each outbreak. The results revealed a prediction accuracy of approximately 80%: only 7 of 35 outbreaks were inconsistent in the number of single-linkage clusters or types. Moreover, the trends in maximum intraoutbreak allelic discrepancy (normalized by scheme size) for the 35 outbreaks in Set B between LmScheme_370 and Lm-cgMLST (the original cgMLST containing 1701 loci) were similar. This suggests that the discriminatory power of LmScheme_370 was equal to that of Lm-cgMLST for detecting different outbreaks.

In addition, as the use of GBG analysis increases, so does the need for storing large quantities of allelic profiles, thereby leading to potential storage burdens. Furthermore, the larger the allelic profile database grows, the longer the time taken to yield query search results. Therefore, a reduced scheme, such as that developed in the present study, can help decrease the maintenance cost and improve query search time.

## Conclusions

In summary, the XGBoost feature selection method has excellent potential to select the most important loci from large typing schemes and retains the discriminatory power to distinguish between outbreaks. Reducing the number of sampled loci by approximately 80% through feature selection may reduce false discrepancies by approximately 80% if errors are distributed evenly throughout the genome sequence. This approach can be applied to schemes and bacteria other than the *L*. *monocytogenes* cgMLST scheme. The increasing use of GBG comparison methodologies in genomic epidemiology makes achieving accurate data interpretation vital. Our locus reduction approach provides an easily applicable means of overcoming the false discrepancy problem and can thus help in genomic epidemiology. Our study targeted only one of the potential problems of cgMLST usage. With the rising popularity of the cgMLST approach, future studies should more comprehensively evaluate potential problems and develop solutions.

## Supporting information

S1 Table List of Set A. (PDF)

S2 Table List of Set B. (PDF)

S3 Table The description of LmScheme_370. (PDF)

S4 Table Demonstration of the specious discrepency caused from different experimental settings for the three tested *L*. *monocytogenes* genomes based on Lm-cgMLST scheme. (PDF)

S5 Table Demonstration of the specious discrepency caused from different experimental settings for the three tested *L*. *monocytogenes* genomes based on LmScheme_370. (PDF)

S1 Fig A single-linkage dendrogram of Set B calculated based on LmScheme_370 with CT types and labeled outbreak clusters. (PDF)
