# Supplementary material for: A machine learning-based typing scheme refinement for Listeria monocytogenes core genome multilocus sequence typing with high discriminatory power for common source outbreak tracking
Source: PLoS One. 2021 Nov 19;16(11):e0260293. doi: 10.1371/journal.pone.0260293 (PMC8604304; doi:10.1371/journal.pone.0260293)
Supplement: S1 Table — (PDF) [file pone.0260293.s001.pdf]

S1 Table. List of Set A.

AccNo	Metadata
NC_018589.1	1966
NC_018592.1	1924 UK, rabbit
NC_022568.1	1924 UK, rabbit
NC_003210.1	1924 UK, rabbit
NZ_CP013287.1	1931 United States or New Zealand animal
NZ_CP007160.1	1935 UK, clinical
NC_018588.1	1935 UK, clinical
NC_017529.1	1950 Netherlands, cheese
NC_018586.1	1956 United States, clinical
NC_018591.1	1966, clinical
NC_018587.1	1967, chinchilla
SRR1814389	1976 France Anjou outbreak, clinical
NC_021825.1	1981 Canada coleslaw outbreak, clinical
SRR1814363	1981 Canada coleslaw outbreak, clinical
SRR1814363	1993 United States, animal
SRR3707731	1981 Canada coleslaw outbreak, food
SRR1814366	1981 UK, clinical
SRR1814368	1983 Boston, MA, milk outbreak, clinical
SRR3707880	1983 Boston, MA, milk outbreak, clinical
CM001159.1	1983 Boston, MA, milk outbreak, clinical
NC_019556.1	1983¬°V1987 Switzerland cheese outbreak, clinical
SRR1814365	1983¬°V1987 Switzerland cheese outbreak, clinical
NZ_CP009242.1	1985 Australia, sheep
NC_002973.6	1985 California cheese outbreak, clinical
SRR1814334	1985 California cheese outbreak, clinical
NC_018593.1	1986 Austria, cheese
SRR1814376	1987 Pennsylvania outbreak, clinical
SRR1814364	1987¬°V1989 UK p?t? outbreak, clinical
SRR1814378	1987¬°V1989 UK p?t? outbreak, clinical
SRR3707715	1987¬°V1989 UK p?t? outbreak, food
SRR3707727	1987¬°V1989 UK p?t? outbreak, food
SRR3707866	1989 United States, animal
NC_021824.1	1989 United States, cow
NC_021838.1	1994 Illinois chocolate milk gastroenteritis outbreak
SRR3707865	1994 United States, animal
NC_021837.1	1996 New York, animal
SRR1814374	1996 U.S. sporadic case, clinical
SRR3707716	1996 United States, clinical
NZ_HG813247.1	1996¬°V1999 smoked salmon, persistent isolate
NC_017547.1	1998 Finland butter outbreak
SRR1814362	1998 U.S. multistate hot dog outbreak, clinical
SRR3707879	1998 U.S. multistate hot dog outbreak, clinical
SRR1815437	1998 U.S. multistate hot dog outbreak, clinical
SRR1814399	1998 U.S. multistate hot dog outbreak, food
SRR3707886	1998 U.S. multistate hot dog outbreak, food
SRR1815440	1998 U.S. multistate hot dog outbreak, food
SRR3707884	1998 U.S. multistate hot dog outbreak, food
SRR3707885	1998 U.S. multistate hot dog outbreak, food
SRR1815438	1998 U.S. multistate hot dog outbreak, food
SRR3707729	1998 United States, clinical
NC_021823.1	1999 New York, meat
SRR1814375	1999 United States, food
SRR1814387	1999 United States, food
NC_012488.1	1999¬°V2000 France pork rillettes outbreak, clinical
NC_017545.1	2000 U.S. multistate turkey deli meat outbreak, clinical
SRR1814386	2000 U.S. sporadic case, clinical
SRR1814380	2000 United States, environment
SRR1814384	2000 United States, environment
SRR1814383	2000 United States, food
NC_021839.1	2002 U.S. multistate turkey deli meat outbreak, clinical
SRR3707894	2002 U.S. multistate turkey deli meat outbreak, clinical
SRR3707893	2002 U.S. multistate turkey deli meat outbreak, clinical

SRR1815439	2002 U.S. multistate turkey deli meat outbreak, clinical
SRR3707726	2002 U.S. multistate turkey deli meat outbreak, clinical
NC_021827.1	2002 U.S. multistate turkey deli meat outbreak, environment
NC_021830.2	2002 U.S. multistate turkey deli meat outbreak, environment
SRR3707728	2002 U.S. multistate turkey deli meat outbreak, environment
NC_021840.1	2002 U.S. multistate turkey deli meat outbreak, food
SRR1814333	2002 U.S. multistate turkey deli meat outbreak, food
SRR3707892	2002 U.S. multistate turkey deli meat outbreak, food
SRR3707876	2002 United States, environment
SRR1814388	2002 United States, environment
SRR1814340	2004 United States, environment
NZ_CP013723.1	2005 Switzerland cheese outbreak, clinical
NZ_CP013722.1	2005 Switzerland cheese outbreak, clinical
NZ_CP009258.1	2006 Switzerland, clinical
NC_013766.2	2008 Canada deli meat outbreak, clinical
NC_013768.1	2008 Canada deli meat outbreak, clinical
NZ_CP013919.1	2008 Italy outbreak, clinical
NZ_CP010346.1	2009 Italy, clinical
NZ_CP007688.1	2010 Louisiana head cheese outbreak, clinical
NZ_CP007689.1	2010 Texas celery outbreak, clinical
SRR2054281	2010¬°V2015 U.S. multistate ice cream outbreak, food
SRR2078889	2010¬°V2015 U.S. multistate ice cream outbreak, food
SRR2054168	2010¬°V2015 U.S. multistate ice cream outbreak, food
SRR2054250	2010¬°V2015 U.S. multistate ice cream outbreak, food
SRR1998952	2010¬°V2015 U.S. multistate ice cream outbreak, food
SRR1982133	2010¬°V2015 U.S. multistate ice cream outbreak, food
SRR1998966	2010¬°V2015 U.S. multistate ice cream outbreak, food
NZ_CP009897.1	2011 China, sheep
NZ_CP013724.1	2011 Switzerland ham outbreak, clinical
NZ_CP007686.1	2011 U.S. multistate cantaloupe outbreak, clinical
NZ_CP007684.1	2011 U.S. multistate cantaloupe outbreak, clinical
NZ_CP007685.1	2011 U.S. multistate cantaloupe outbreak, clinical
NZ_CP007687.1	2011 U.S. multistate cantaloupe outbreak, clinical
SRR1908952	2011 U.S. multistate cantaloupe outbreak, environment
SRR3644931	2011 U.S. multistate cantaloupe outbreak, environmental
SRR3644928	2011 U.S. multistate cantaloupe outbreak, environmental
SRR1908945	2011 U.S. multistate cantaloupe outbreak, food
NZ_CP014250.1	2013 U.S. Hispanic-style cheese outbreak, food
SRR1182223	2013 U.S. Hispanic-style cheese outbreak, food
SRR1181522	2013 U.S. Hispanic-style cheese outbreak, food
SRR1378358	2013 U.S. Hispanic-style cheese outbreak, food
SRR955385	2013 U.S. multistate French-style cheese outbreak, food
SRR955387	2013 U.S. multistate French-style cheese outbreak, food
NZ_CP007600.1	2013 U.S. multistate French-style cheese outbreak, food
NZ_CP011004.1	2013¬°V2014 Switzerland salad outbreak, clinical
SRR1619552	2014 U.S. multistate cheese outbreak, environment
SRR1635202	2014 U.S. multistate cheese outbreak, environment
SRR1980624	2014 U.S. multistate cheese outbreak, food
SRR1980616	2014 U.S. multistate cheese outbreak, food
SRR1571519	2014 U.S. multistate stone fruit outbreak, environment
NZ_CP012021.1	2014 U.S. multistate stone fruit outbreak, food
SRR1553779	2014 U.S. multistate stone fruit outbreak, food
SRR1553906	2014 U.S. multistate stone fruit outbreak, food
SRR1553882	2014 U.S. multistate stone fruit outbreak, food
SRR1571543	2014 U.S. stone fruit recall, environment
SRR1556287	2014 U.S. stone fruit recall, food
SRR1553871	2014 U.S. stone fruit recall, food
NZ_CP014252.1	2014 U.S. stone fruit recall, food
SRR1767799	2014¬°V2015 U.S. multistate caramel apple outbreak, environment
SRR1767800	2014¬°V2015 U.S. multistate caramel apple outbreak, environment
SRR1763864	2014¬°V2015 U.S. multistate caramel apple outbreak, food
SRR1763833	2014¬°V2015 U.S. multistate caramel apple outbreak, food
SRR1770498	2014¬°V2015 U.S. multistate caramel apple outbreak, food
SRR1770500	2014¬°V2015 U.S. multistate caramel apple outbreak, food

SRR1763846	2014¬°V2015 U.S. multistate caramel apple outbreak, food
SRR1763839	2014¬°V2015 U.S. multistate caramel apple outbreak, food
NZ_CP014261.1	2014¬°V2016 Italy outbreak
SRR3147176	2015¬°V2016 U.S. multistate packaged salad outbreak, food
SRR3147177	2015¬°V2016 U.S. multistate packaged salad outbreak, food
SRR3147179	2015¬°V2016 U.S. multistate packaged salad outbreak, food
SRR3147180	2015¬°V2016 U.S. multistate packaged salad outbreak, food
SRR1814370	Canada, clinical
NC_018642.1	Cheese
NC_018585.1	Chicken
NC_017537.1	China, cow milk
NC_021826.1	Environment
SRR1814352	Europe, food
NZ_CP007492.1	Food
NZ_HG813249.1	Ireland cheese facility, persistent strain
NC_017728.1	Massachusetts sporadic case, clinical
NZ_CP013289.1	
NZ_CP011397.1	
SRR1814377	
NZ_CP007210.1	NAd
NC_018590.1	Pre-1961 England, chicken
NZ_CP013286.1	Pre-1961 England, chicken
NZ_CP013285.1	Pre-1961 UK, poultry
NC_018584.1	Pre-1961 United States, sheep
NC_017544.1	Pre-1968, derivative of 10403 from U.S. clinical case
SRR3707717	UK sporadic case, clinical
NC_017546.1	UK, clinical
SRR3707730	United States
NC_011660.1	United States, catfish
SRR3707734	United States, clinical
SRR1814400	United States, food
SRR3707878	United States, food
SRR1818112	United States, monkey
