# Supplementary material for: A machine learning-based typing scheme refinement for Listeria monocytogenes core genome multilocus sequence typing with high discriminatory power for common source outbreak tracking
Source: PLoS One. 2021 Nov 19;16(11):e0260293. doi: 10.1371/journal.pone.0260293 (PMC8604304; doi:10.1371/journal.pone.0260293)
Supplement: S2 Table — (PDF) [file pone.0260293.s002.pdf]

S2 Table. List of Set B.

Run	Outbreak_Id
SRR961948	0908MLGX6-1
SRR961949	0908MLGX6-1
SRR961953	0908MLGX6-1
SRR961956	0908MLGX6-1
SRR961958	0908MLGX6-1
SRR945179	1005NYGX6-1
SRR954533	1005NYGX6-1
SRR954534	1005NYGX6-1
SRR954535	1005NYGX6-1
SRR954536	1005NYGX6-1
SRR954537	1005NYGX6-1
SRR954538	1005NYGX6-1
SRR954539	1005NYGX6-1
SRR954540	1005NYGX6-1
SRR954541	1005NYGX6-1
SRR954542	1005NYGX6-1
SRR954543	1005NYGX6-1
SRR954544	1005NYGX6-1
SRR954546	1005NYGX6-1
SRR954548	1005NYGX6-1
SRR954549	1005NYGX6-1
SRR954550	1005NYGX6-1
SRR954551	1005NYGX6-1
SRR954552	1005NYGX6-1
SRR954553	1005NYGX6-1
SRR954554	1005NYGX6-1
SRR1068557	1207PAGX6-1
SRR1068558	1207PAGX6-1
SRR1068559	1207PAGX6-1
SRR1068560	1207PAGX6-1
SRR1068561	1207PAGX6-1
SRR1068562	1207PAGX6-1
SRR1068563	1207PAGX6-1
SRR1068564	1207PAGX6-1
SRR1068565	1207PAGX6-1
SRR1068566	1207PAGX6-1
SRR1068567	1207PAGX6-1
SRR1068568	1207PAGX6-1
SRR1068569	1207PAGX6-1
SRR1068570	1207PAGX6-1
SRR1068571	1207PAGX6-1
SRR1068572	1207PAGX6-1
SRR1068573	1207PAGX6-1
SRR1068574	1207PAGX6-1
SRR1068575	1207PAGX6-1
SRR1068576	1207PAGX6-1
SRR1068577	1207PAGX6-1
SRR1068578	1207PAGX6-1
SRR1068579	1207PAGX6-1
SRR1068580	1207PAGX6-1
SRR1068581	1207PAGX6-1
SRR1068582	1207PAGX6-1
SRR1068583	1207PAGX6-1
SRR1068584	1207PAGX6-1
SRR1112171	1207PAGX6-1
SRR1016574	1301MLGX6-1
SRR1016576	1301MLGX6-1
SRR1016579	1301MLGX6-1
SRR1016589	1301MLGX6-1
SRR1016597	1301MLGX6-1
SRR1016601	1301MLGX6-1
SRR1016922	1301MLGX6-1

SRR1021891	1301MLGX6-1
SRR1021947	1301MLGX6-1
SRR1027073	1301MLGX6-1
SRR1030273	1301MLGX6-1
SRR1039786	1301MLGX6-1
SRR1039790	1301MLGX6-1
SRR1039791	1301MLGX6-1
SRR1039792	1301MLGX6-1
SRR1039793	1301MLGX6-1
SRR1039795	1301MLGX6-1
SRR1039796	1301MLGX6-1
SRR1039797	1301MLGX6-1
SRR1039800	1301MLGX6-1
SRR1043165	1301MLGX6-1
SRR1043175	1301MLGX6-1
SRR1043176	1301MLGX6-1
SRR1195645	1301MLGX6-1
SRR945167	1301MLGX6-1
SRR946032	1301MLGX6-1
SRR974850	1301MLGX6-1
SRR974883	1301MLGX6-1
SRR988748	1301MLGX6-1
SRR988749	1301MLGX6-1
SRR1001027	1307MLGX6-1
SRR1016591	1307MLGX6-1
SRR1016594	1307MLGX6-1
SRR1016605	1307MLGX6-1
SRR1016609	1307MLGX6-1
SRR1016925	1307MLGX6-1
SRR1021894	1307MLGX6-1
SRR1021895	1307MLGX6-1
SRR1021948	1307MLGX6-1
SRR1021953	1307MLGX6-1
SRR1027086	1307MLGX6-1
SRR1030279	1307MLGX6-1
SRR1030280	1307MLGX6-1
SRR1041609	1307MLGX6-1
SRR1041615	1307MLGX6-1
SRR1112081	1307MLGX6-1
SRR946033	1307MLGX6-1
SRR957726	1307MLGX6-1
SRR972392	1307MLGX6-1
SRR972406	1307MLGX6-1
SRR988736	1307MLGX6-1
SRR1005719	1307MLGX6-1,2
SRR1112220	1307MLGX6-1,2
SRR972407	1307MLGX6-1,2
SRR974858	1307MLGX6-1,2
SRR1005715	1307MLGX6-2
SRR1016586	1307MLGX6-2
SRR1016599	1307MLGX6-2
SRR1022668	1307MLGX6-2
SRR1027088	1307MLGX6-2
SRR1039803	1307MLGX6-2
SRR1041621	1307MLGX6-2
SRR1041629	1307MLGX6-2
SRR945161	1307MLGX6-2
SRR959025	1307MLGX6-2
SRR974878	1307MLGX6-2
SRR974886	1307MLGX6-2
SRR988747	1307MLGX6-2
SRR1016585	1307MNGX6-1
SRR1016603	1307MNGX6-1
SRR1041624	1307MNGX6-1

SRR945150	1307MNGX6-1
SRR945151	1307MNGX6-1
SRR945156	1307MNGX6-1
SRR945163	1307MNGX6-1
SRR945181	1307MNGX6-1
SRR959028	1307MNGX6-1
SRR972394	1307MNGX6-1
SRR974877	1307MNGX6-1
SRR1005718	1308ILGX6-1
SRR945147	1308ILGX6-1
SRR945152	1308ILGX6-1
SRR945153	1308ILGX6-1
SRR974876	1308ILGX6-1
SRR974881	1308ILGX6-1
SRR1027093	1308MDGX6-1
SRR1112195	1308MDGX6-1
SRR1193826	1308MDGX6-1
SRR1195657	1308MDGX6-1
SRR1195661	1308MDGX6-1
SRR1195691	1308MDGX6-1
SRR1198951	1308MDGX6-1
SRR1041623	1308MLGX6-1
SRR1112130	1308MLGX6-1
SRR972390	1308MLGX6-1
SRR972395	1308MLGX6-1
SRR1005716	1308MLGX6-2
SRR972391	1308MLGX6-2
SRR1005499	1309MLGX6-1
SRR1016565	1309MLGX6-1
SRR1021893	1309MLGX6-1
SRR953801	1309MLGX6-1
SRR988737	1309MLGX6-1
SRR1016564	1309MLGX6-2WGS
SRR1016598	1309MLGX6-2WGS
SRR1022670	1309MLGX6-2WGS
SRR1027067	1309MLGX6-2WGS
SRR1027070	1309MLGX6-2WGS
SRR1027082	1309MLGX6-2WGS
SRR945166	1309MLGX6-2WGS
SRR945173	1309MLGX6-2WGS
SRR974869	1309MLGX6-2WGS
SRR988750	1309MLGX6-2WGS
SRR1001049	1310VAGX6-1
SRR1016568	1310VAGX6-1
SRR1030272	1310VAGX6-1
SRR988731	1310VAGX6-1
SRR1016572	1311MAGX6-1
SRR1016588	1311MAGX6-1
SRR1027069	1311MAGX6-1
SRR1027078	1311MAGX6-1
SRR1033761	1311MAGX6-1
SRR1041625	1311MAGX6-1
SRR1043169	1311MAGX6-1
SRR945148	1311MAGX6-1
SRR945149	1311MAGX6-1
SRR945180	1311MAGX6-1
SRR972399	1311MAGX6-1
SRR1030283	1311MLGX6-1
SRR1030284	1311MLGX6-1
SRR1030333	1311MLGX6-1
SRR1030334	1311MLGX6-1
SRR1030335	1311MLGX6-1
SRR1030336	1311MLGX6-1
SRR1030337	1311MLGX6-1

SRR1030338	1311MLGX6-1
SRR1030339	1311MLGX6-1
SRR1030340	1311MLGX6-1
SRR1030342	1311MLGX6-1
SRR1030343	1311MLGX6-1
SRR1030344	1311MLGX6-1
SRR1030345	1311MLGX6-1
SRR1030346	1311MLGX6-1
SRR1030348	1311MLGX6-1
SRR1030349	1311MLGX6-1
SRR1033767	1311MLGX6-1
SRR1033768	1311MLGX6-1
SRR1033769	1311MLGX6-1
SRR1033770	1311MLGX6-1
SRR1039787	1311MLGX6-1
SRR1039789	1311MLGX6-1
SRR1043163	1311MLGX6-1
SRR1068290	1311MLGX6-1
SRR1068317	1311MLGX6-1
SRR1068342	1311MLGX6-1
SRR1101347	1311MLGX6-1
SRR1101497	1311MLGX6-1
SRR1101501	1311MLGX6-1
SRR945169	1311MLGX6-1
SRR974887	1311MLGX6-1
SRR988742	1311MLGX6-1
SRR1016924	1311MLGX6-2WGS
SRR1021943	1311MLGX6-2WGS
SRR1021949	1311MLGX6-2WGS
SRR1022666	1311MLGX6-2WGS
SRR1027066	1311MLGX6-2WGS
SRR1027071	1311MLGX6-2WGS
SRR1032218	1311MLGX6-2WGS
SRR1039801	1311MLGX6-2WGS
SRR1101646	1311MLGX6-2WGS
SRR945158	1311MLGX6-2WGS
SRR972409	1311MLGX6-2WGS
SRR1027076	1312LACGX6-1
SRR1112191	1312LACGX6-1
SRR974882	1312LACGX6-1
SRR1005504	1312MLGX6-1
SRR1005723	1312MLGX6-1
SRR1021945	1312MLGX6-1
SRR1021951	1312MLGX6-1
SRR1022667	1312MLGX6-1
SRR1043167	1312MLGX6-1
SRR1112177	1312MLGX6-1
SRR1112186	1312MLGX6-1
SRR945146	1312MLGX6-1
SRR974879	1312MLGX6-1
SRR974853	1403MLGX6-1WGS
SRR988732	1403MLGX6-1WGS
SRR1619219	1411RIGX6-1WGS
SRR1619223	1411RIGX6-1WGS
SRR1695787	1411RIGX6-1WGS
SRR1657624	1411RIGX6-1WGS
SRR1695803	1411RIGX6-1WGS
SRR1695804	1411RIGX6-1WGS
SRR1695813	1411RIGX6-1WGS
SRR1779446	1411RIGX6-1WGS
SRR1752636	1411RIGX6-1WGS
SRR1027104	1411RIGX6-1WGS
ERR1599820	DK-CC224-2014
ERR1599821	DK-CC224-2014

ERR1599822	DK-CC224-2014
ERR1599823	DK-CC224-2014
ERR1599824	DK-CC224-2014
ERR1599825	DK-CC224-2014
ERR1599826	DK-CC224-2014
ERR1599827	DK-CC224-2014
ERR1599828	DK-CC224-2014
ERR1599829	DK-CC224-2014
ERR1599830	DK-CC224-2014
ERR1599831	DK-CC224-2014
ERR1599819	DK-CC224-2014
ERR1599857	DK-CC224-2014
ERR1599856	DK-CC224-2014
ERR1599855	DK-CC224-2014
ERR1599853	DK-CC224-2014
ERR1599851	DK-CC224-2014
ERR1599849	DK-CC224-2014
ERR1599848	DK-CC224-2014
ERR1599847	DK-CC224-2014
ERR1599846	DK-CC224-2014
ERR1599845	DK-CC224-2014
ERR1599844	DK-CC224-2014
ERR1599854	DK-CC224-2014
ERR1599843	DK-CC224-2014
ERR1599842	DK-CC224-2014
ERR1599841	DK-CC224-2014
ERR1599858	DK-CC224-2014
ERR1599840	DK-CC224-2014
ERR1599839	DK-CC224-2014
ERR1599850	DK-CC224-2014
ERR1599838	DK-CC224-2014
ERR1599837	DK-CC224-2014
ERR1599836	DK-CC224-2014
ERR1599835	DK-CC224-2014
ERR1599834	DK-CC224-2014
ERR1599833	DK-CC224-2014
ERR1599832	DK-CC224-2014
ERR1599859	DK-CC224-2014
ERR1599804	DK-CC224-2014
ERR1599852	DK-CC224-2014
ERR1599678	MF-b
ERR1599679	MF-b
ERR1599725	MF-c
ERR1599726	MF-c
ERR1599683	MF-d
ERR1599700	MF-d
ERR1599743	MF-e
ERR1599744	MF-e
ERR1599717	Mom-a
ERR1599718	Mom-a
ERR1599719	Mom-a
ERR1599720	Mom-a
ERR1599721	Mom-a
ERR1599722	Mom-a
ERR1599723	Mom-a
ERR1599724	Mom-a
ERR1599727	Mom-a
SRR4052270	cluster 1
SRR4052183	cluster 1
SRR4052274	cluster 10
SRR4052336	cluster 10
SRR4052339	cluster 10
SRR4052205	cluster 11
SRR4052086	cluster 11

SRR4052266	cluster 5
SRR4052342	cluster 5
SRR4052158	cluster 5
SRR4052243	cluster 5
SRR4052171	cluster 5
SRR4052217	cluster 7
SRR4052185	cluster 7
SRR4052201	cluster 7
SRR4052124	cluster 8
SRR4052271	cluster 8
SRR4052126	cluster 8
SRR4052344	cluster 8
SRR4052107	cluster 8
SRR4052123	cluster 8
SRR4052106	cluster 9
SRR4052343	cluster 9
SRR4052096	cluster 9
