# Supplementary material for: A machine learning-based typing scheme refinement for Listeria monocytogenes core genome multilocus sequence typing with high discriminatory power for common source outbreak tracking
Source: PLoS One. 2021 Nov 19;16(11):e0260293. doi: 10.1371/journal.pone.0260293 (PMC8604304; doi:10.1371/journal.pone.0260293)
Supplement: S3 Table — (PDF) [file pone.0260293.s003.pdf]

S3 Table. The description of LmScheme_370.

Locus_id (1,701)	Gene	Product	SHAP value
lmo2571		nicotinamidase	9.46
lmo0227		hypothetical protein	6.50
lmo0005	recF	recombination protein F	4.11
lmo0003		hypothetical protein	3.72
lmo1174	eutA	ethanolamine utilization protein EutA	3.46
lmo1525		recombination protein RecJ	3.09
lmo2165		Crp/Fnr family transcriptional regulator	2.92
lmo2538	upp	uracil phosphoribosyltransferase	2.89
lmo2546	thrC	threonine synthase	2.86
lmo2720		acetate-CoA ligase	2.84
lmo0300		phospho-beta-galactosidase	2.71
lmo0006	gyrB	DNA gyrase subunit B	2.56
lmo0007	gyrA	DNA gyrase subunit A	2.54
lmo2211	hemH	ferrochelatase	2.44
lmo0973	dltB	DltB protein for D-alanine esterification of lipoteichoic acid and wall teichoic acid	2.41
lmo0292		heat-shock protein htrA serine protease	2.34
lmo1511		hypothetical protein	2.13
lmo0676	fliP	flagellar biosynthesis protein FliP	2.10
lmo2386		hypothetical protein	1.93
lmo0369		hypothetical protein	1.82
lmo0210	ldh	L-lactate dehydrogenase	1.78
lmo0153		zinc ABC transporter substrate-binding protein	1.69
lmo1762		hypothetical protein	1.64
lmo0386		IolD protein	1.63
lmo0401		alpha-mannosidase	1.51
lmo0152		peptide ABC transporter substrate-binding protein	1.46
lmo0164		DNA replication intiation control protein YabA	1.46
lmo0105		chitinase B	1.36
lmo1861		hypothetical protein	1.32
lmo0540		penicillin-binding protein	1.32
lmo1615	trmB	tRNA (guanine-N(7)-)-methyltransferase	1.32
lmo1498		O-methyltransferase	1.23
lmo0974	dltA	D-alanine--poly(phosphoribitol) ligase subunit 1	1.23
lmo1481	holA	DNA polymerase III subunit delta	1.21
lmo2229		penicillin-binding protein	1.20
lmo1912		histidine kinase	1.16
lmo2692		hypothetical protein	1.15
lmo2362		amino acid antiporter	1.14
lmo2235		NADH oxidase	1.12
lmo2471		NADPH dehydrogenase	1.12
lmo1482	comEC	competence protein ComEC	1.10
lmo1663	ansB	asparagine synthetase	1.10
lmo0392		hypothetical protein	1.08
lmo0233		DNA repair protein RadA	1.06
lmo0272		hypothetical protein	1.05
lmo1734		glutamate synthase large subunit	1.01
lmo0685		flagellar motor protein MotA	0.97
lmo0219		hypothetical protein	0.93
lmo1177		carboxysome structural protein EutL	0.90
lmo2349		amino acid ABC transporter substrate-binding protein	0.90
lmo2581		hypothetical protein	0.90
lmo2842		LacI family transcriptional regulator	0.90
lmo2161		hypothetical protein	0.89
lmo1244		phosphoglycerate mutase	0.88
lmo1051	def	peptide deformylase	0.88
lmo0231		ATP:guanido phosphotransferase	0.86
lmo0847		glutamine ABC transporter	0.85
lmo2492		hypothetical protein	0.84
lmo0851		hypothetical protein	0.82
lmo1235		aspartate kinase	0.82

lmo0786		ACP phosphodiesterase	0.81
lmo1231		DNA polymerase beta	0.80
lmo1860		methionine sulfoxide reductase A	0.80
lmo1720		PTS lichenan transporter subunit IIB	0.80
lmo1896	asnC	asparaginyl-tRNA synthetase	0.78
lmo2083		hypothetical protein	0.78
lmo1377	lisR	two-component response regulator	0.78
lmo0288		two-component sensor histidine kinase	0.77
lmo0572		hypothetical protein	0.76
lmo0908		hypothetical protein	0.76
lmo1689		A/G-specific adenine glycosylase	0.75
lmo2531	atpA	ATP synthase F0F1 subunit alpha	0.75
lmo1834	pyrDII	dihydroorotate dehydrogenase electron transfer subunit	0.72
lmo1318		hypothetical protein	0.72
lmo0511		hypothetical protein	0.70
lmo2535	atpB	ATP synthase F0F1 subunit A	0.70
lmo0904		hypothetical protein	0.69
lmo2346		amino acid ABC transporter ATP-binding protein	0.69
lmo1824	priA	primosome assembly protein PriA	0.69
lmo2834		oxidoreductase	0.68
lmo0267		hypothetical protein	0.68
lmo0008		cardiolipin synthase	0.68
lmo1583	tpx	thiol peroxidase	0.68
lmo1165		ethanolamine utilization protein EutE	0.67
lmo2050		excinuclease ABC subunit A	0.67
lmo2449		exoribonuclease RNase-R	0.66
lmo1579		alanine dehydrogenase	0.65
lmo0027		PTS beta-glucoside transporter subunit IIABC	0.65
lmo2504		cell wall-binding protein	0.64
lmo0013	qoxA	AA3-600 quinol oxidase subunit II	0.64
lmo1848		metal ABC transporter permease	0.64
lmo1695		hypothetical protein	0.64
lmo1810		fatty acid biosynthesis transcriptional regulator	0.64
lmo0556		phosphoglycerate mutase	0.63
lmo1450		DEAD/DEAH box helicase	0.63
lmo1952	lysA	diaminopimelate decarboxylase	0.63
lmo2017		hypothetical protein	0.62
lmo1697		hypothetical protein	0.62
lmo1341		competence protein ComG	0.62
lmo0030		hypothetical protein	0.62
lmo0349		hypothetical protein	0.61
lmo2391		hypothetical protein	0.61
lmo1721		transcriptional regulator	0.61
lmo1016	gbuC	glycine/betaine ABC transporter substrate-binding protein	0.60
lmo2668		transcriptional antiterminator BglG	0.60
lmo1534		L-lactate dehydrogenase	0.58
lmo1210		hypothetical protein	0.58
lmo1466		hypothetical protein	0.57
lmo0629		hypothetical protein	0.56
lmo1637		hypothetical protein	0.55
lmo1499		hypothetical protein	0.55
lmo0866		ATP-dependent RNA helicase	0.55
lmo2213		hypothetical protein	0.52
lmo0488		LysR family transcriptional regulator	0.51
lmo1863		hypothetical protein	0.50
lmo1957	fhuG	ferrichrome ABC transporter permease	0.50
lmo0759		hypothetical protein	0.50
lmo0910		hypothetical protein	0.50
lmo1316	cdsA	phosphatidate cytidylyltransferase	0.49
lmo1050		hypothetical protein	0.49
lmo0822		transcriptional regulator	0.48
lmo0382		transcriptional regulator	0.48
lmo1446	zurM	metal (zinc) transport protein (ABC transporter, permease)	0.47
lmo0642		hypothetical protein	0.47

lmo0947		hypothetical protein	0.47
lmo0681		flagellar biosynthesis regulator FlhF	0.47
lmo0696	flgD	flagellar basal body rod modification protein	0.47
lmo2070		hypothetical protein	0.47
lmo1759	pcrA	ATP-dependent DNA helicase	0.47
lmo0888		hypothetical protein	0.47
lmo2171		MFS transporter	0.47
lmo2451	secG	preprotein translocase subunit SecG	0.47
lmo0515		hypothetical protein	0.46
lmo1811		ATP-dependent DNA helicase RecG	0.46
lmo1792	trmD	tRNA (guanine-N(1)-)-methyltransferase	0.46
lmo0574		beta-glucosidase	0.46
lmo1495		hypothetical protein	0.45
lmo2699		hypothetical protein	0.45
lmo0517		phosphoglycerate mutase	0.45
lmo0521		6-phospho-beta-glucosidase	0.45
lmo0707	fliD	flagellar capping protein FliD	0.45
lmo2094		L-fuculose-phosphate aldolase	0.45
lmo0256		hypothetical protein	0.44
lmo0650		hypothetical protein	0.44
lmo0883		hypothetical protein	0.44
lmo2770		bifunctional glutamate--cysteine ligase/glutathione synthetase	0.43
lmo2376		peptidyl-prolyl cis-trans isomerase	0.43
lmo1702		glutathione transferase	0.43
lmo0795		hypothetical protein	0.42
lmo1699		chemotaxis protein	0.42
lmo2096		PTS galacticol transporter subunit IIC	0.42
lmo0843		hypothetical protein	0.42
lmo1269		type I signal peptidase	0.41
lmo2247		oxidoreductase	0.41
lmo1246		ATP-dependent RNA helicase	0.41
lmo1337		hypothetical protein	0.40
lmo0190	ipk	4-diphosphocytidyl-2-C-methyl-D-erythritol kinase	0.40
lmo0407		hypothetical protein	0.40
lmo0355		fumarate reductase subunit A	0.40
lmo1963		hypothetical protein	0.40
lmo2263		hypothetical protein	0.40
lmo0214	mfd	transcription-repair coupling factor	0.40
lmo1245		hypothetical protein	0.40
lmo2392		hypothetical protein	0.39
lmo2172		propionate CoA-transferase	0.39
lmo2035	murG	UDP-diphospho-muramoylpentapeptide beta-N-acetylglucosaminyltransferase	0.39
lmo0102		hypothetical protein	0.38
lmo1918		hypothetical protein	0.38
lmo0242		hypothetical protein	0.37
lmo0022		PTS fructose transporter subunit IIB	0.37
lmo1585		peptidase	0.37
lmo1287	parC	DNA topoisomerase IV subunit A	0.37
lmo2506	ftsX	cell division protein FtsX	0.37
lmo2488	uvrA	excinuclease ABC subunit A	0.36
lmo1168	AckA2	acetate kinase	0.36
lmo0645		amino acid transporter	0.36
lmo0025		phosphoheptose isomerase	0.36
lmo0898		hypothetical protein	0.36
lmo0690	flaA	flagellin	0.36
lmo1438		penicillin-binding protein	0.36
lmo0266		transcriptional regulator	0.36
lmo2126		maltogenic amylase	0.36
lmo2516		hypothetical protein	0.36
lmo0931		lipoate protein ligase A	0.35
lmo2454		hypothetical protein	0.35
lmo1166		NADPH-dependent butanol dehydrogenase	0.35
lmo1897	aspB	aspartate aminotransferase	0.34
lmo1283		LacX protein	0.34

lmo0960		protease	0.34
lmo0859		sugar ABC transporter substrate-binding protein	0.33
lmo1950		hypothetical protein	0.32
lmo0679	flhB	flagellar biosynthesis protein FlhB	0.32
lmo0687		hypothetical protein	0.32
lmo1240		hypothetical protein	0.31
lmo0807		spermidine/putrescine ABC transporter ATP-binding protein	0.31
lmo0605		hypothetical protein	0.30
lmo2482	lgt	prolipoprotein diacylglyceryl transferase	0.30
lmo1493		oligopeptidase	0.30
lmo2759		hypothetical protein	0.29
lmo0977		hypothetical protein	0.28
lmo0697	flgE	flagellar hook protein FlgE	0.28
lmo0611	acpD	azoreductase	0.27
lmo0241		hypothetical protein	0.27
lmo0785		transcriptional regulator	0.27
lmo0963		heat shock protein HtpX	0.26
lmo1346	comGB	competence protein ComGB	0.26
lmo0258	rpoB	DNA-directed RNA polymerase subunit beta	0.25
lmo2014		sugar hydrolase	0.25
lmo2089		lipase	0.24
lmo2785	kat	catalase	0.24
lmo1809	plsX	glycerol-3-phosphate acyltransferase PlsX	0.24
lmo0485		hypothetical protein	0.24
lmo1096	guaA	GMP synthase	0.24
lmo0486	rpmF	50S ribosomal protein L32	0.24
lmo0212		hypothetical protein	0.24
lmo0346		triosephosphate isomerase	0.24
lmo1476	hemN	coproporphyrinogen III oxidase	0.24
lmo0024		PTS mannose transporter subunit IID	0.24
lmo2354		hypothetical protein	0.24
lmo2796		transcriptional regulator	0.24
lmo1317		1-deoxy-D-xylulose 5-phosphate reductoisomerase	0.24
lmo0995		hypothetical protein	0.24
lmo0688		hypothetical protein	0.24
lmo2839		sugar ABC transporter substrate-binding protein	0.24
lmo1895	dnaD	chromosome replication initiation protein	0.24
lmo1635		hypothetical protein	0.24
lmo0606		MarR family transcriptional regulator	0.24
lmo0372		beta-glucosidase	0.24
lmo0035		glucosamine--fructose-6-phosphate aminotransferase	0.24
lmo1239		nucleoside-triphosphatase	0.24
lmo2769		ABC transporter ATP-binding protein	0.24
lmo0728		riboflavin kinase / FAD synthase	0.24
lmo2665		PTS galacticol transporter subunit IIC	0.24
lmo0762		ATP/GTP-binding protein	0.24
lmo2585		hypothetical protein	0.24
lmo0783		PTS mannose transporter subunit IIB	0.24
lmo1345		competence protein ComGC	0.24
lmo1374		branched-chain alpha-keto acid dehydrogenase subunit E2	0.24
lmo2433		acetylesterase	0.24
lmo0282		hypothetical protein	0.24
lmo2412		hypothetical protein	0.24
lmo0930		hypothetical protein	0.24
lmo1020		hypothetical protein	0.24
lmo1935		protein-tyrosine/serine phosphatase	0.23
lmo2854		sporulation protein SpoJ	0.23
lmo2823		sporulation protein SpoOJ	0.23
lmo1173		two-component sensor histidine kinase	0.23
lmo0441		D-alanyl-D-alanine carboxypeptidase	0.23
lmo0021		PTS fructose transporter subunit IIA	0.23
lmo1313	pyrH	uridylate kinase	0.23
lmo0683		chemotaxis protein CheR	0.23
lmo1788		transcriptional regulator	0.23

lmo2037	mraY	phospho-N-acetylmuramoyl-pentapeptide- transferase	0.23
lmo2724		hypothetical protein	0.23
lmo1887		hypothetical protein	0.23
lmo0593		formate transporter	0.23
lmo1938	rpsA	30S ribosomal protein S1	0.23
lmo0177	metS	methionyl-tRNA synthetase	0.23
lmo1475	hrcA	heat-inducible transcription repressor	0.23
lmo0951		hypothetical protein	0.23
lmo1372		branched-chain alpha-keto acid dehydrogenase subunit E1	0.23
lmo1807	fabG	3-ketoacyl-ACP reductase	0.23
lmo2695		dihydroxyacetone kinase subunit DhaK	0.23
lmo1150		transcriptional regulator PocR	0.23
lmo2404		hypothetical protein	0.23
lmo1479	lepA	GTP-binding protein LepA	0.23
lmo2857		hypothetical protein	0.23
lmo2766		RpiR family transcriptional regulator	0.23
lmo1472	dnaJ	molecular chaperone DnaJ	0.23
lmo2570		hypothetical protein	0.23
lmo0594	metX	homoserine O-acetyltransferase	0.23
lmo1282		hypothetical protein	0.23
lmo2599		hypothetical protein	0.23
lmo0519		multidrug resistance protein	0.23
lmo2455	eno	phosphopyruvate hydratase	0.23
lmo1199	cbiH	precorrin-3B C17-methyltransferase	0.23
lmo0192		PurR family transcriptional regulator	0.23
lmo2372		ABC transporter ATP-binding protein	0.23
lmo1285		hypothetical protein	0.23
lmo2483		HPr kinase/phosphorylase	0.23
lmo0240		hypothetical protein	0.23
lmo0188	ksgA	dimethyladenosine transferase	0.23
lmo0480		transcriptional regulator	0.23
lmo2539	glyA	serine hydroxymethyltransferase	0.23
lmo0221		pantothenate kinase	0.23
lmo1010		LysR family transcriptional regulator	0.23
lmo0932		hypothetical protein	0.23
lmo2088		transcriptional regulator	0.23
lmo2419		ABC transporter ATP-binding protein	0.23
lmo2545	thrB	homoserine kinase	0.23
lmo2662		ribose 5-phosphate epimerase	0.23
lmo1379		sporulation protein SpoJ	0.23
lmo1151		PduA protein	0.23
lmo2012		hypothetical protein	0.23
lmo1899	dinG	bifunctional ATP-dependent DNA helicase/DNA polymerase III subunit epsilon	0.23
lmo0427		PTS fructose transporter subunit IIB	0.23
lmo0203	mpl	Zinc metalloproteinase precursor	0.22
lmo0201	plcA	phosphatidylinositol-specific phospholipase c	0.22
lmo2056		hypothetical protein	0.22
lmo0269		transporter	0.22
lmo2489	uvrB	excinuclease ABC subunit B	0.22
lmo0437		hypothetical protein	0.22
lmo1930		heptaprenyl diphosphate synthase subunit II	0.22
lmo1592		thiamine biosynthesis protein ThiI	0.22
lmo1367		arginine repressor ArgR	0.22
lmo2738		hemolysin	0.22
lmo0814		oxidoreductase	0.22
lmo1844	lspA	lipoprotein signal peptidase	0.22
lmo1541		hypothetical protein	0.22
lmo0966		hypothetical protein	0.22
lmo0547		DeoR family transcriptional regulator	0.22
lmo2099		transcriptional antiterminator	0.22
lmo0988	prfC	peptide chain release factor 3	0.22
lmo0281		hypothetical protein	0.22
lmo2397		NifU protein	0.22
lmo1601		general stress protein	0.22

lmo0243	sigH	RNA polymerase factor sigma-70	0.22
lmo2496		phosphate ABC transporter ATP-binding protein	0.22
lmo0699	fliM	flagellar motor switch protein FliM	0.22
lmo2698		RpiR family transcriptional regulator	0.22
lmo0626		hypothetical protein	0.22
lmo1859		methionine sulfoxide reductase B	0.22
lmo0055	purA	adenylosuccinate synthetase	0.22
lmo1320	polC	DNA polymerase III PolC	0.22
lmo1440		hypothetical protein	0.22
lmo0375		hypothetical protein	0.22
lmo1181		cobalamin adenosyl transferase	0.21
lmo2062		copper transporter	0.21
lmo0135		peptide ABC transporter substrate-binding protein	0.21
lmo1171	pduQ	NADPH-dependent butanol dehydrogenase	0.21
lmo0857		carboxylesterase	0.21
lmo2457	tpiA	triosephosphate isomerase	0.21
lmo2103	eutD	phosphotransacetylase	0.21
lmo1370		butyrate kinase	0.21
lmo1349		glycine dehydrogenase subunit 1	0.21
lmo0232	clpC	endopeptidase Clp ATP-binding chain C	0.21
lmo0926		TetR family transcriptional regulator	0.20
lmo1547	mreC	rod shape-determining protein MreC	0.20
lmo2013		hypothetical protein	0.16
lmo2389		NADH dehydrogenase	0.16
lmo0524		sulfate transporter	0.15
lmo1357		acetyl-CoA carboxylase biotin carboxylase subunit	0.13
lmo1628	trpB	tryptophan synthase subunit beta	0.13
lmo0959		undacaprenyl-phosphate N-acetylglucosaminyltransferase	0.13
lmo0377		hypothetical protein	0.12
lmo2238		MFS transporter	0.12
lmo2381		monovalent cation/H+ antiporter subunit D	0.11
lmo2600	cbiO	cobalt ABC transporter ATP-binding subunit	0.10
lmo1331	pnpA	polynucleotide phosphorylase	0.10
lmo0400		PTS fructose transporter subunit IIC	0.10
lmo0829	nifJ	pyruvate-flavodoxin oxidoreductase	0.09
lmo1521		N-acetylmuramoyl-L-alanine amidase	0.09
lmo1520	hisS	histidyl-tRNA synthetase	0.09
lmo2659		ribulose-phosphate 3-epimerase	0.08
lmo2353		Na+/H+ antiporter	0.08
lmo1178		carboxysome structural protein	0.07
lmo0797		hypothetical protein	0.07
lmo0824		hypothetical protein	0.06
lmo0002	dnaN	DNA polymerase III subunit beta	0.06
lmo0316		hydroxyethylthiazole kinase	0.06
lmo1706		transporter	0.06
lmo2651		PTS mannitol transporter subunit IIA	0.05
lmo0863		hypothetical protein	0.05
lmo0202	hly	listeriolysin O precursor	0.04
lmo1578		X-Pro dipeptidase	0.04
lmo1234	uvrC	excinuclease ABC subunit C	0.04
lmo0162		DNA polymerase III subunit delta'	0.03
lmo0802		hypothetical protein	0.03
lmo0892	rsbU	serine phosphatase	0.03
lmo1739		amino acid ABC transporter ATP-binding protein	0.01
