# Supplementary material for: A machine learning-based typing scheme refinement for Listeria monocytogenes core genome multilocus sequence typing with high discriminatory power for common source outbreak tracking
Source: PLoS One. 2021 Nov 19;16(11):e0260293. doi: 10.1371/journal.pone.0260293 (PMC8604304; doi:10.1371/journal.pone.0260293)
Supplement: S4 Table — (PDF) [file pone.0260293.s004.pdf]

S4 Table. Demonstration of the specious discrepency caused from different experimental settings for the three tested *L. monocytogenes* genomes based on Lm-cgMLST scheme.

**GCF_016775745.1_cgMLST**	20x_HiSeq_skesa	20x_MiSeq_skesa	50x_HiSeq_skesa	50x_MiSeq_skesa	20x_HiSeq_spades	20x_MiSeq_spades	50x_HiSeq_spades	50x_MiSeq_spades
20x_HiSeq_skesa	0	33	8	8	8	8	8	8
20x_MiSeq_skesa	33	0	25	25	25	25	25	25
50x_HiSeq_skesa	8	25	0	0	0	0	0	0
50x_MiSeq_skesa	8	25	0	0	0	0	0	0
20x_HiSeq_spades	8	25	0	0	0	0	0	0
20x_MiSeq_spades	8	25	0	0	0	0	0	0
50x_HiSeq_spades	8	25	0	0	0	0	0	0
50x_MiSeq_spades	8	25	0	0	0	0	0	0
**GCF_016802645.1_cgMLST**	20x_HiSeq_skesa	20x_MiSeq_skesa	50x_HiSeq_skesa	50x_MiSeq_skesa	20x_HiSeq_spades	20x_MiSeq_spades	50x_HiSeq_spades	50x_MiSeq_spades
20x_HiSeq_skesa	0	31	13	13	13	14	13	13
20x_MiSeq_skesa	31	0	18	18	18	17	18	18
50x_HiSeq_skesa	13	18	0	0	0	1	0	0
50x_MiSeq_skesa	13	18	0	0	0	1	0	0
20x_HiSeq_spades	13	18	0	0	0	1	0	0
20x_MiSeq_spades	14	17	1	1	1	0	1	1
50x_HiSeq_spades	13	18	0	0	0	1	0	0
50x_MiSeq_spades	13	18	0	0	0	1	0	0
**GCF_905219385.1_cgMLST**	20x_HiSeq_skesa	20x_MiSeq_skesa	50x_HiSeq_skesa	50x_MiSeq_skesa	20x_HiSeq_spades	20x_MiSeq_spades	50x_HiSeq_s[ades	50x_MiSeq_spades
20x_HiSeq_skesa	0	33	8	8	7	8	8	8
20x_MiSeq_skesa	33	0	25	25	26	25	25	25
50x_HiSeq_skesa	8	25	0	0	1	0	0	0
50x_MiSeq_skesa	8	25	0	0	1	0	0	0
20x_HiSeq_spades	7	26	1	1	0	1	1	1
20x_MiSeq_spades	8	25	0	0	1	0	0	0
50x_HiSeq_spades	8	25	0	0	1	0	0	0
50x_MiSeq_spades	8	25	0	0	1	0	0	0
