# Supplementary material for: A machine learning-based typing scheme refinement for Listeria monocytogenes core genome multilocus sequence typing with high discriminatory power for common source outbreak tracking
Source: PLoS One. 2021 Nov 19;16(11):e0260293. doi: 10.1371/journal.pone.0260293 (PMC8604304; doi:10.1371/journal.pone.0260293)
Supplement: S5 Table — (PDF) [file pone.0260293.s005.pdf]

S5 Table. Demonstration of the specious discrepency caused from different experimental settings for the three tested *L. monocytogenes* genomes based on LmScheme_370.

**GCF_016775745.1_370-scheme**	20x_HiSeq_skesa	20x_MiSeq_skesa	50x_HiSeq_skesa	50x_MiSeq_skesa	20x_HiSeq_spades	20x_MiSeq_spades	50x_HiSeq_spades	50x_MiSeq_spades
20x_HiSeq_skesa	0	6	2	2	2	2	2	2
20x_MiSeq_skesa	6	0	4	4	4	4	4	4
50x_HiSeq_skesa	2	4	0	0	0	0	0	0
50x_MiSeq_skesa	2	4	0	0	0	0	0	0
20x_HiSeq_spades	2	4	0	0	0	0	0	0
20x_MiSeq_spades	2	4	0	0	0	0	0	0
50x_HiSeq_spades	2	4	0	0	0	0	0	0
50x_MiSeq_spades	2	4	0	0	0	0	0	0
**GCF_016802645.1_370-scheme**	20x_HiSeq_skesa	20x_MiSeq_skesa	50x_HiSeq_skesa	50x_MiSeq_skesa	20x_HiSeq_spades	20x_MiSeq_spades	50x_HiSeq_sades	50x_MiSeq_spades
20x_HiSeq_skesa	0	7	5	5	5	5	5	5
20x_MiSeq_skesa	7	0	2	2	2	2	2	2
50x_HiSeq_skesa	5	2	0	0	0	0	0	0
50x_MiSeq_skesa	5	2	0	0	0	0	0	0
20x_HiSeq_sades	5	2	0	0	0	0	0	0
20x_MiSeq_spades	5	2	0	0	0	0	0	0
50x_HiSeq_spades	5	2	0	0	0	0	0	0
50x_MiSeq_spades	5	2	0	0	0	0	0	0
**GCF_905219385.1_370-scheme**	20x_HiSeq_skesa	20x_MiSeq_skesa	50x_HiSeq_skesa	50x_MiSeq_skesa	20x_HiSeq_spades	20x_MiSeq_spades	50x_HiSeq_spades	50x_MiSeq_spades
20x_HiSeq_skesa	0	2	1	1	0	1	1	1
20x_MiSeq_skesa	2	0	1	1	2	1	1	1
50x_HiSeq_skesa	1	1	0	0	1	0	0	0
50x_MiSeq_skesa	1	1	0	0	1	0	0	0
20x_HiSeq_spades	0	2	1	1	0	1	1	1
20x_MiSeq_spades	1	1	0	0	1	0	0	0
50x_HiSeq_spades	1	1	0	0	1	0	0	0
50x_MiSeq_spades	1	1	0	0	1	0	0	0
