# Supplementary material for: A machine learning-based typing scheme refinement for Listeria monocytogenes core genome multilocus sequence typing with high discriminatory power for common source outbreak tracking
Source: PLoS One. 2021 Nov 19;16(11):e0260293. doi: 10.1371/journal.pone.0260293 (PMC8604304; doi:10.1371/journal.pone.0260293)
Supplement: S1 Fig — (PDF) [file pone.0260293.s006.pdf]

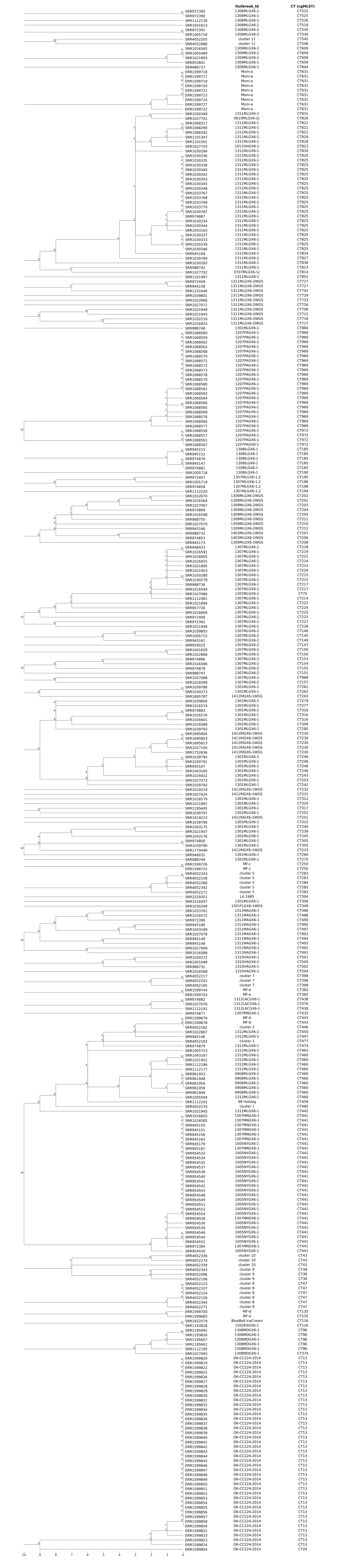

S1 Figure. A single-linkage dendrogram of Set B calculated based on LmScheme_370 with CT types and labeled outbreak clusters.
